# Objective Severity, Pain and Range-of-Motion Limitation in Breast Cancer-Related Upper-Limb Lymphedema: Associations with Patient-Reported Burden

**DOI:** 10.3390/jcm15166345

**Published:** 2026-08-17

**Authors:** Emine Çetin Duru, Mehmet Adam, Pınar Doruk Analan

**Affiliations:** 1 Department of Physical Medicine & Rehabilitation, Adana City Education & Research Hospital, 01230 Adana, Turkey; 2 Department of Physical Medicine & Rehabilitation, Adana MedLine Hospital, 01170 Adana, Turkey; mehmetadam@yahoo.com; 3 Department of Physical Medicine & Rehabilitation, Dr. Turgut Noyan Training and Research Center, Faculty of Medicine, Baskent University, 06790 Adana, Turkey; doruk.pinar@gmail.com

**Keywords:** breast cancer lymphedema, patient-reported outcome measures, quality of life, rehabilitation

## Abstract

**Background/Objectives**: Breast cancer-related upper-limb lymphedema (BCRL) can impair arm function and quality of life, while objective measures may not fully reflect patient-perceived burden. This study examined associations of objective severity, pain intensity, and shoulder range-of-motion (ROM) limitation with disability and lymphedema-specific quality of life in women with BCRL. **Methods**: This cross-sectional analysis included 110 women with clinically diagnosed BCRL. Objective severity was assessed using International Society of Lymphology stage, circumferential measurements, and water displacement volumetry. Pain was evaluated with a visual analog scale. Disability was assessed using the Quick Disabilities of the Arm, Shoulder and Hand questionnaire (QuickDASH), and lymphedema-specific quality of life using the Quality of Life Measure for Limb Lymphedema-Arm (LYMQOL-Arm). Analyses included nonparametric comparisons, Spearman correlations, and exploratory multivariable regression. **Results**: Higher lymphedema stage was associated with greater circumferential and volumetric differences and worse QuickDASH, LYMQOL function, and LYMQOL appearance scores. Objective severity measures correlated with QuickDASH, LYMQOL function, and LYMQOL appearance, but not with pain or LYMQOL symptoms. Shoulder ROM limitation was associated with higher pain, worse QuickDASH, higher LYMQOL function, appearance, and symptom scores, and lower global quality of life. In regression analyses, pain intensity, volumetric difference, and shoulder ROM limitation remained associated with QuickDASH, whereas pain intensity was the main factor associated with global quality of life. **Conclusions**: Objective severity measures mainly reflected anatomical, functional, and appearance-related burden but did not fully capture pain, symptoms, or global quality of life. Multidimensional assessment combining limb measurements, pain evaluation, shoulder mobility assessment, and patient-reported outcomes may support a more comprehensive clinical evaluation of women with BCRL.

## 1. Introduction

Breast cancer-related upper-limb lymphedema (BCRL) is a chronic and potentially progressive complication that may occur after breast cancer surgery and adjuvant treatments [1]. Its incidence varies widely, ranging from approximately 4% to 30%, depending on the diagnostic criteria, measurement methods, and follow-up intervals used across studies [2]. BCRL may present with a constellation of symptoms, including upper-limb swelling, pain, heaviness, tightness, limited range of motion (ROM), and weakness. These symptoms may impair upper-limb function, cause difficulties in activities of daily living, and reduce quality of life [3,4,5].

BCRL is not merely an increase in limb volume. As lymphatic stasis progresses, tissue fibrosis, dermal thickening, mobility restrictions, functional decline, and psychosocial burden may become more apparent [5,6]. In clinical practice, the International Society of Lymphology (ISL) staging system is used to determine the clinical stage of lymphedema, whereas circumferential measurements and volumetric assessments are commonly used to quantify its anatomical and objective severity [7,8]. Imaging-based approaches, including ultrasonographic assessment of dermal, subcutaneous, and fascial tissue changes, may provide additional information on tissue-level involvement in limb lymphedema [9]. However, these objective indicators may not fully capture the impact of the condition on perceived symptoms, daily functioning, and quality of life. Objective measurements and patient-reported outcome measures (PROMs) related to pain, mobility limitations, and functional difficulties define distinct but overlapping domains of lymphedema burden [10,11].

Although treatment-related factors, body mass index, and demographic characteristics have been extensively discussed in relation to the development and severity of lymphedema, the clinical factors that contribute to the discrepancy between objective severity and patient-perceived burden remain insufficiently understood. Previous studies have examined selected components of this relationship, including associations between objective lymphedema measurements, upper-limb disability, symptom burden, and quality of life [11,12,13]. However, fewer studies have evaluated objective severity measures together with pain intensity, clinically assessed shoulder ROM limitation, generic upper-limb disability, and separate domains of lymphedema-specific quality of life within the same cohort. In particular, pain intensity and shoulder ROM limitation may affect upper-limb use and daily activities, thereby increasing perceived functional impairment beyond what can be explained by limb volume alone. Therefore, in the management of BCRL, a more comprehensive clinical approach may be achieved by evaluating clinically relevant dimensions such as pain, shoulder ROM limitation, upper-limb disability, and lymphedema-specific quality of life, rather than focusing solely on objective severity. Beyond limb swelling, chronic lymphatic stasis has been associated with progressive tissue fibrosis, which may in turn contribute to the physical and functional changes observed in BCRL [14]. Persistent pain in breast cancer survivors, including those with lymphedema, has also been linked to central sensitization processes that may amplify symptom perception independently of tissue-level severity [15].

The aim of this study was to examine the associations of objective lymphedema severity, pain intensity, and shoulder ROM limitation with upper-limb dysfunction, symptom burden, and quality of life, as assessed using the QuickDASH and LYMQOL-Arm scales, in women with clinically established BCRL. Understanding these associations may help clarify which components of clinical assessment are most closely related to patient-reported disability and lymphedema-specific quality-of-life burden in this population.

## 2. Materials and Methods

### 2.1. Study Design and Participants

This cross-sectional study included 110 women with breast cancer-related upper-limb lymphedema. The study investigated the relationships between objective lymphedema severity, pain, shoulder range-of-motion (ROM) limitation, functional disability, and lymphedema-specific quality of life.

Data were collected prospectively between January 2016 and June 2017 at the Physical Medicine and Rehabilitation Unit of Başkent University Adana Dr. Turgut Noyan Training and Research Hospital, using a structured clinical and patient-reported assessment protocol. Patients meeting the eligibility criteria for this cross-sectional study and presenting to the outpatient clinic during this period were enrolled consecutively.

Women aged 31 to 75 years with clinically diagnosed breast cancer-related upper-limb lymphedema were included. All participants were able to complete the questionnaires independently.

Patients meeting any of the following criteria were not included in the study: actively receiving chemotherapy or radiotherapy at the time of assessment; clinically unstable disease; general health status precluding study participation; neurological disease affecting upper-limb function; inflammatory rheumatic disease; clinically significant pre-existing shoulder pathology unrelated to breast cancer treatment; a history of shoulder-mobility-affecting surgery within the preceding three months; or inability to complete the questionnaires independently, including insufficient Turkish literacy to do so.

### 2.2. Demographic and Clinical Variables

Demographic and clinical data were collected using a structured patient information form. Variables recorded included age, education level, occupation, dominant hand, body mass index (BMI), side of lymphedema, chemotherapy history, radiotherapy history, lymph node dissection history and type, duration of swelling, smoking status, and other disease-related characteristics. Time elapsed since breast cancer surgery was also recorded, in months. Cancer-related clinical information was recorded based on patient report and cross-checked against available medical records, such as pathology reports and surgical records; values that could not be verified were coded as missing. BMI was analyzed both as a continuous variable and categorically using a cutoff value of 30 kg/m^2^.

### 2.3. Clinical and Objective Lymphedema Assessment

All participants underwent a standardized clinical examination performed by the same physical medicine and rehabilitation physician with relevant knowledge and experience in lymphedema assessment throughout the study. The presence of motor deficit, Stemmer sign, axillary cording, and pitting edema was recorded as present or absent.

Active shoulder range of motion was assessed bilaterally using a standard goniometer, in six directions: flexion, extension, abduction, adduction, internal rotation, and external rotation. A reduction of at least 10° in any of these directions on the affected side, compared with the clinically unaffected contralateral side, was considered a range-of-motion limitation [16], consistent with prior evaluations of shoulder morbidity after breast cancer treatment [17]. Passive range of motion was also examined as part of the routine clinical assessment, primarily to help distinguish joint-based restriction (e.g., adhesive capsulitis) from active/functional limitation, but was not systematically recorded as a separate study variable.

Lymphedema stage was determined according to the International Society of Lymphology classification and categorized as stage I, stage II, or stage III.

Objective lymphedema severity was assessed using circumferential and volumetric measurements. Circumferential measurements were obtained bilaterally using a measuring tape at five predefined anatomical levels: the metacarpophalangeal joint level, wrist, 10 cm distal to the olecranon, 10 cm proximal to the olecranon, and 20 cm proximal to the olecranon. The olecranon was used as the anatomical reference point for the proximal and distal arm measurements. These measurement sites are based on the simplified four-point method (dorsum of the hand, wrist, 10 cm distal to the olecranon, and 10 cm proximal to the olecranon) described at the 2006 London International Lymphoedema Framework meeting [18]. A fifth site, 20 cm proximal to the olecranon, was added to also capture proximal arm involvement; water displacement volumetry was extended to the same level for consistency. In participants with unilateral lymphedema, interlimb circumferential and volumetric differences were calculated by subtracting the measurement of the clinically unaffected limb from that of the affected limb. In participants with bilateral involvement, the limb with more clinically advanced findings, based on ISL stage and clinical signs such as the Stemmer sign, was designated as the affected/index limb. This approach was applied to one participant in the present cohort, who had asymmetric bilateral lymphedema with one limb classified as ISL Stage I and the other as ISL Stage II. For this participant, circumferential and volumetric differences were calculated by subtracting the measurements of the Stage I limb from those of the Stage II limb. The mean circumferential difference was calculated by averaging the differences obtained from the five measurement sites [18].

**Figure 1:**
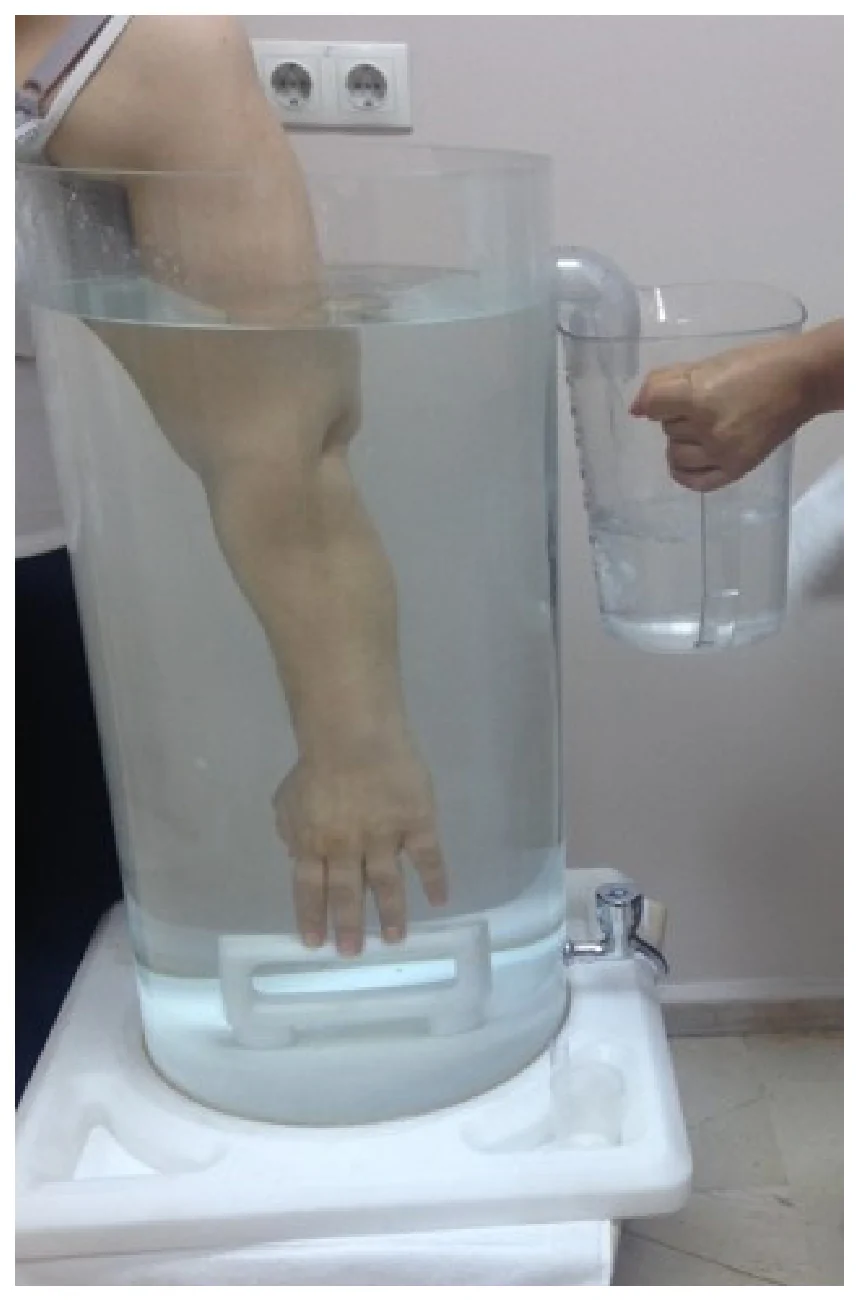
Water displacement volumetry method used to assess upper-limb volume. The limb was immersed in a water-filled volumetric container, and the displaced water volume was recorded in milliliters.

Upper-limb volume was assessed bilaterally using the water displacement method, which provides a simple and low-cost estimate of limb volume in clinical practice. Each limb was immersed sequentially in a water-filled volumetric container, and the displaced water volume was recorded in milliliters [7] (Figure 1).

### 2.4. Patient-Reported Outcome Measures

Pain intensity was assessed using a 10-cm visual analog scale (VAS), ranging from 0 (no pain) to 10 (worst imaginable pain), based on participants’ rating of the pain they had experienced in the lymphedematous limb over the preceding week.

Upper-extremity disability was evaluated using the Quick Disabilities of the Arm, Shoulder and Hand questionnaire (QuickDASH). Scores range from 0 to 100, with higher scores indicating greater disability [19]. The validated Turkish version of the QuickDASH was used [20].

Lymphedema-specific quality of life was assessed using the Quality of Life Measure for Limb Lymphedema-Arm (LYMQOL-Arm). The questionnaire includes function, appearance, symptom, and mood domains, together with a global quality-of-life item. Higher scores in the domain subscales indicate greater impairment, whereas higher scores on the global quality-of-life item indicate better overall quality of life [21]. The validated Turkish version of the LYMQOL-Arm was used [22].

### 2.5. Statistical Analysis

No a priori sample size calculation was performed for the present analysis. The sample size was determined by the number of eligible and consenting women with clinically diagnosed BCRL who were consecutively enrolled during the study period. Subgroup comparisons, correlation analyses, and multivariable regression models were therefore considered exploratory.

Analyses were organized into main and secondary/exploratory categories. The main analyses addressed the central aim of the study: the associations of objective lymphedema severity measures, shoulder ROM limitation, and Stemmer sign with pain, disability, and lymphedema-specific quality of life. Secondary/exploratory analyses included supportive subgroup comparisons and sensitivity analyses conducted in response to reviewer comments. To account for the risk of false-positive findings arising from multiple comparisons in the main bivariate analyses, the Benjamini–Hochberg false discovery rate (FDR) procedure was applied.

Statistical analyses were performed using jamovi version 2.7.28. Continuous variables were summarized as mean ± standard deviation or median and interquartile range according to distribution characteristics. Categorical variables were presented as frequencies and percentages.

Normality was assessed using histograms, Q-Q plots, and the Shapiro–Wilk test. Associations between objective lymphedema severity measures and patient-reported outcomes were examined using Spearman correlation analysis.

Group comparisons according to lymphedema stage, BMI category, shoulder ROM limitation, Stemmer sign, and pitting edema were performed using nonparametric methods. The Mann–Whitney U test was used for two-group comparisons, whereas the Kruskal–Wallis test was used for comparisons involving three groups. Significant Kruskal–Wallis results were followed by Dunn–Bonferroni post hoc analyses.

To identify factors associated with upper-extremity disability and global quality of life, multivariable linear regression analyses were performed. Separate models were constructed using QuickDASH and global quality-of-life scores as dependent variables. Age, BMI, pain intensity, volumetric difference, and shoulder ROM limitation were entered as independent variables. Unstandardized coefficients (B) with 95% confidence intervals and standardized regression coefficients (β) with corresponding 95% confidence intervals were reported as obtained from the regression model output. Regression model assumptions were evaluated using residual plots, Q-Q plots, the Shapiro–Wilk test for residual normality, the Breusch–Pagan test for homoscedasticity, Cook’s distance for influential observations, and variance inflation factors for multicollinearity. Statistical significance was set at *p* < 0.05.

## 3. Results

### 3.1. Demographic and Clinical Characteristics

A total of 110 women with breast cancer-related upper-limb lymphedema were included in the study. The mean age was 54.1 ± 10.0 years, and the mean body mass index was 31.9 ± 5.8 kg/m^2^. The median duration of lymphedema was 9.0 months (Q1–Q3: 1.3–22.0). Demographic and clinical characteristics of the study population are summarized in Table 1.

Median time since breast cancer surgery was 29.0 months (IQR: 15.0–50.0). No significant correlations were found between time since surgery and any patient-reported outcome (all *p* > 0.26). A sensitivity analysis additionally adjusting for lymphedema duration and time since surgery showed that the associations of pain intensity, volumetric difference, and shoulder ROM limitation with QuickDASH remained materially unchanged; neither duration variable was independently associated with QuickDASH or global quality of life (both *p* > 0.43).

### 3.2. Objective Measurements and Patient-Reported Outcomes

Objective lymphedema severity was assessed using volumetric difference, mean circumferential difference, and lymphedema stage. Patient-reported outcomes included pain intensity (VAS), upper-extremity disability (QuickDASH), and lymphedema-specific quality of life assessed by the LYMQOL function, appearance, symptom, and mood domains together with the global quality-of-life score. Descriptive statistics for these measures are presented in Table 2.

A post hoc control analysis based on dominant-side involvement showed no significant differences between patients whose dominant limb was affected and those whose non-dominant limb was affected, in volumetric difference (*p* = 0.501), mean circumferential difference (*p* = 0.250), QuickDASH (*p* = 0.519), or global quality of life (*p* = 0.348).

### 3.3. Objective Measurements and Patient-Reported Outcomes According to Lymphedema Stage

Comparisons according to lymphedema stage are presented in Table 3. 

**Figure 2:**
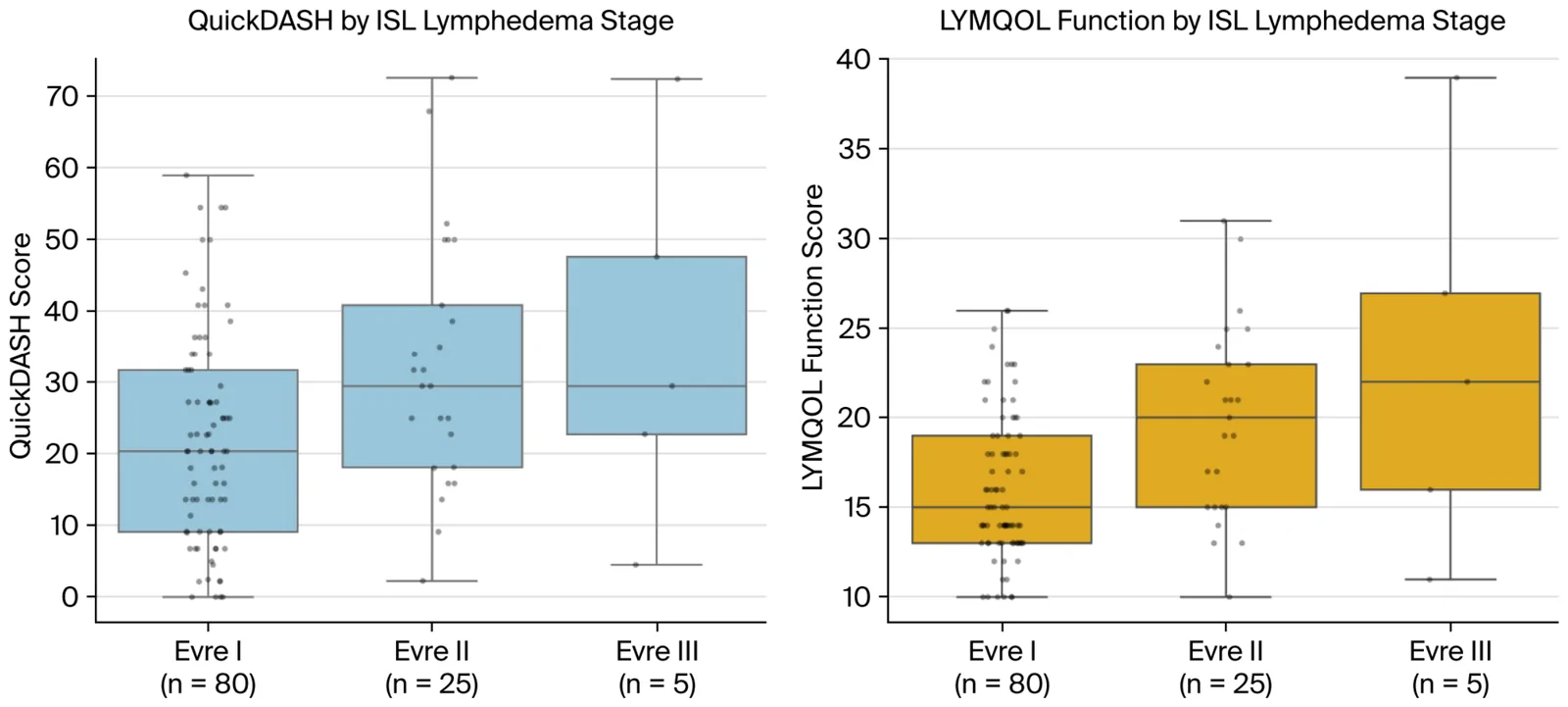
QuickDASH and LYMQOL Function scores according to ISL lymphedema stage. Boxes represent the interquartile range (Q1–Q3), horizontal lines indicate the median, and individual data points are overlaid. Note the limited number of participants in the Stage III subgroup (n = 5).

Significant differences were observed among lymphedema stages for QuickDASH (*p* = 0.020), LYMQOL function (*p* = 0.003), LYMQOL appearance (*p* < 0.001), mean circumferential difference (*p* < 0.001), and volumetric difference (*p* < 0.001) (Figure 2).

No statistically significant differences were found among lymphedema stages for VAS (*p* = 0.737), LYMQOL symptom scores (*p* = 0.530), LYMQOL mood scores (*p* = 0.268), or global quality-of-life scores (*p* = 0.052).

Post hoc Dunn–Bonferroni analyses demonstrated significant differences between Stage I and Stage II for QuickDASH (adjusted *p* = 0.026), LYMQOL function (adjusted *p* = 0.006), and LYMQOL appearance scores (adjusted *p* < 0.001). For mean circumferential difference and volumetric difference, significant differences were observed between Stage I and Stage II (both adjusted *p* < 0.001) and between Stage I and Stage III (both adjusted *p* < 0.001). No significant differences were identified between Stage II and Stage III for any of these variables (all adjusted *p* > 0.05).

To further evaluate whether the absence of significant differences between Stage II and Stage III reflected a true similarity in disease burden or limited statistical power, we performed a sensitivity analysis combining Stage II and Stage III participants into a single group (n = 30) and comparing them with Stage I (n = 80) using the Mann–Whitney U test. Under this two-group comparison, global quality of life, which had not reached significance in the original three-group comparison (*p* = 0.052), became statistically significant (*p* = 0.015), while findings for the remaining outcomes were consistent with the original three-group analysis (Supplementary Table S2).

### 3.4. Associations Between Objective Lymphedema Severity Measures and Patient-Reported Outcomes

Correlations between objective measures of lymphedema severity and patient-reported outcomes are presented in Table 4. 

Lymphedema stage was significantly correlated with QuickDASH (ρ = 0.266, *p* = 0.005), LYMQOL function (ρ = 0.322, *p* < 0.001), LYMQOL appearance (ρ = 0.416, *p* < 0.001), and global quality-of-life scores (ρ = −0.231, *p* = 0.015).

This Spearman correlation result (*p* = 0.015) is consistent with the Stage I versus combined Stage II + III sensitivity analysis reported in Section 3.3 (*p* = 0.015). The apparent discrepancy with the original three-group Kruskal–Wallis comparison (*p* = 0.052) may therefore be partly related to limited statistical power arising from the small Stage III subgroup, rather than indicating conflicting evidence. No significant correlations were observed between lymphedema stage and VAS (ρ = 0.074, *p* = 0.442), LYMQOL symptom scores (ρ = 0.108, *p* = 0.262), or LYMQOL mood scores (ρ = 0.150, *p* = 0.117).

Volumetric difference was significantly correlated with QuickDASH (ρ = 0.225, *p* = 0.018), LYMQOL function (ρ = 0.248, *p* = 0.009), and LYMQOL appearance scores (ρ = 0.404, *p* < 0.001). No significant correlations were found between volumetric difference and VAS (ρ = −0.039, *p* = 0.687), LYMQOL symptom scores (ρ = 0.080, *p* = 0.408), LYMQOL mood scores (ρ = 0.044, *p* = 0.648), or global quality-of-life scores (ρ = −0.093, *p* = 0.334).

The mean circumferential difference was significantly correlated with QuickDASH (ρ = 0.292, *p* = 0.002), LYMQOL function (ρ = 0.307, *p* = 0.001), LYMQOL appearance (ρ = 0.456, *p* < 0.001), LYMQOL mood scores (ρ = 0.259, *p* = 0.006), and global quality-of-life scores (ρ = −0.273, *p* = 0.004). No significant correlations were observed between mean circumferential difference and VAS (ρ = −0.038, *p* = 0.693) or LYMQOL symptom scores (ρ = 0.141, *p* = 0.142).

### 3.5. Clinical Examination Findings and Patient-Reported Outcomes

Shoulder ROM limitation was associated with significantly higher VAS (*p* = 0.005), QuickDASH (*p* = 0.001), LYMQOL function (*p* < 0.001), LYMQOL appearance (*p* = 0.009), and LYMQOL symptom scores (*p* = 0.002), as well as lower global quality-of-life scores (*p* = 0.009). No significant difference was observed for LYMQOL mood scores (*p* = 0.142) (Table 5).

Patients with a positive Stemmer sign had significantly higher QuickDASH (*p* < 0.001), LYMQOL function (*p* < 0.001), LYMQOL appearance (*p* < 0.001), and LYMQOL mood scores (*p* = 0.009), together with lower global quality-of-life scores (*p* = 0.002), compared with those with a negative Stemmer sign. No significant differences were observed for VAS (*p* = 0.155) or LYMQOL symptom scores (*p* = 0.107).

In addition, patients with a positive Stemmer sign had significantly greater volumetric differences (median 950 mL [Q1–Q3: 650–1150] vs. 270 mL [Q1–Q3: 215–410], *p* < 0.001) and mean circumferential differences (median 3.4 cm [Q1–Q3: 1.7–4.7] vs. 1.1 cm [Q1–Q3: 0.9–1.5], *p* < 0.001).

No significant differences were found between patients with and without pitting edema regarding VAS, QuickDASH, LYMQOL function, appearance, symptom, mood, or global quality-of-life scores. All *p* values were greater than 0.05.

To evaluate whether the associations observed for Stemmer sign reflected confounding by lymphedema severity, we performed multiple linear regression analyses of the form outcome ~ Stemmer sign + volumetric difference. After adjustment for volumetric difference, Stemmer sign remained statistically associated with QuickDASH (B = 13.76, *p* = 0.003), LYMQOL Function (B = 4.34, *p* = 0.002), LYMQOL Mood (B = 3.63, *p* < 0.001), and global quality of life (B = −1.39, *p* = 0.003), whereas its associations with VAS, LYMQOL Appearance, and LYMQOL Symptoms were attenuated to non-significance. These findings suggest that Stemmer sign may provide information beyond limb volume alone for some patient-reported outcomes; however, residual confounding by other indicators of lymphedema severity and treatment-related morbidity cannot be excluded.

### 3.6. BMI and Patient-Reported Outcomes

As a descriptive, clinically oriented comparison based on the widely used 30 kg/m^2^ threshold for obesity, patients were also categorized according to obesity status; BMI was retained as a continuous variable in the multivariable regression models to avoid loss of information. When patients were categorized according to obesity status (BMI ≥ 30 kg/m^2^ vs. <30 kg/m^2^), the obese group demonstrated a significantly greater volumetric difference (median 350 mL vs. 260 mL, *p* = 0.037). No significant difference was observed for mean circumferential difference (*p* = 0.309). Similarly, VAS, QuickDASH, LYMQOL function, appearance, symptom, mood, and global quality-of-life scores did not differ significantly between groups (all *p* > 0.05). As a robustness check, BMI was additionally examined using three WHO obesity classes (<30, n = 39; 30–35, n = 37; >35, n = 34); results were consistent with the two-category analysis (all *p* > 0.37).

### 3.7. Lymph Node Dissection Type and Radiotherapy History

Lymph node dissection type was known for 85 of the 110 participants (sentinel lymph node dissection, n = 13; axillary lymph node dissection, n = 72). No statistically significant differences were observed between these groups for pain, disability, or any LYMQOL domain (all *p* > 0.29; Supplementary Table S3).

Patients were also compared according to radiotherapy history (yes, n = 93 vs. no, n = 17). No significant differences were found in lymphedema stage, QuickDASH, volumetric difference, or mean circumferential difference (all *p* > 0.06). In contrast, LYMQOL Mood (*p* = 0.021) and global quality-of-life scores (*p* = 0.006) differed significantly between groups (Supplementary Table S4).

### 3.8. Multivariable Regression Analyses

The results of the exploratory multivariable regression analyses are presented in Table 6.

In the exploratory multivariable model for QuickDASH scores, higher pain intensity (B = 3.03, 95% CI: 1.81 to 4.24, *p* < 0.001), greater volumetric difference (B = 0.012, 95% CI: 0.005 to 0.019, *p* = 0.001), and the presence of shoulder ROM limitation (B = 8.67, 95% CI: 1.51 to 15.82, *p* = 0.018) were statistically associated with higher disability scores after adjustment for age and BMI. BMI and age were not statistically associated with QuickDASH scores. The model explained 35.2% of the variance in QuickDASH scores, with an adjusted R^2^ of 0.321. In the exploratory model for global quality of life, only pain intensity was statistically associated with lower global quality-of-life scores (B = −0.186, 95% CI: −0.326 to −0.045, *p* = 0.010). The model explained 12.7% of the variance, with an adjusted R^2^ of 0.085, indicating limited explanatory performance (Table 6).

Variance inflation factors ranged from 1.06 to 1.23, indicating no relevant multicollinearity. Residual normality showed mild deviation in both models (QuickDASH: *p* = 0.011; global QoL: *p* = 0.007). The Breusch–Pagan test indicated no heteroscedasticity in the QuickDASH model (*p* = 0.60), whereas heteroscedasticity was detected in the global QoL model (*p* = 0.017). Cook’s distance did not identify any observation with disproportionate influence in either model (maximum D = 0.090). Given the limited explanatory performance and heteroscedasticity of the global QoL model, this model was interpreted as exploratory.

## 4. Discussion

This study showed that objective severity indicators in BCRL were associated with patient-reported functional impairment and quality-of-life burden. However, pain and shoulder ROM limitation appeared to reflect more directly perceived components of the clinical burden. Overall, our findings suggest that objective lymphedema severity and patient-reported outcomes are only incompletely associated, indicating that objective measurement alone does not fully capture the patient-reported burden of BCRL.

The relationship between objective severity measures, such as limb volume and circumference, and patient-reported disease burden, including symptoms, functional limitations, and psychosocial distress, is complex and often discordant. Several studies have shown that objective measurements do not always correspond closely with patient-reported outcomes. For example, some individuals with minimal swelling may report substantial disability, whereas others with more pronounced volume changes may perceive relatively little subjective burden [11,13,23]. Recent research has therefore stressed the necessity of integrated assessment approaches that combine objective and patient-reported tools to better capture the full impact of BCRL on daily life and rehabilitation needs [24,25,26].

In the present study, increasing ISL stage was accompanied by marked increases in mean circumferential difference and volumetric difference, indicating that clinical staging reflected the anatomical severity of limb involvement. However, this increase in objective severity was not mirrored across all patient-reported outcomes. Lymphedema stage was significantly associated with QuickDASH, LYMQOL function, and LYMQOL appearance scores, suggesting that more advanced stages are more closely related to upper-limb functional difficulty and appearance-related burden. This pattern was further supported by the correlation analyses, in which lymphedema stage, volumetric difference, and mean circumferential difference were consistently associated with QuickDASH, LYMQOL function, and LYMQOL appearance scores. These correlations were weak to moderate in magnitude, indicating limited shared variance rather than strong correspondence between objective severity and patient-reported outcomes. The strongest and most consistent associations were observed for LYMQOL appearance, suggesting that visible limb enlargement and asymmetry may be more directly reflected in appearance-related quality-of-life concerns. The particularly strong correlation observed with LYMQOL appearance is consistent with the conceptual proximity between objective limb measurements and appearance-related self-report, and should be regarded as an expected convergent association rather than a novel finding.

To evaluate the clinical relevance of the observed between-group differences, we compared the 9.1-point QuickDASH difference between Stage I and combined Stage II/III lymphedema with the meta-analytic MCID range of 12–15 points recently proposed by Galardini et al. [27]. Because this difference fell below the proposed MCID range, the statistically significant between-group difference in QuickDASH should be interpreted as modest in clinical magnitude. A well-established MCID for the LYMQOL-Arm in the BCRL population could not be identified; therefore, we avoided interpreting LYMQOL score differences against a fixed clinical threshold.

In contrast, neither stage-based comparisons nor correlation analyses showed significant associations between objective severity measures and pain intensity or LYMQOL symptom scores. This finding is clinically relevant because it indicates that anatomical severity does not solely determine the symptom burden in BCRL. Pain, heaviness, tightness, and perceived functional limitation may also be influenced by shoulder dysfunction, treatment-related soft tissue changes, axillary cording, neuropathic mechanisms, and psychosocial factors [28,29]. Similarly, global quality of life showed only weak associations with lymphedema stage and mean circumferential difference and was not significantly associated with volumetric difference. This supports the view that global quality of life is a multidimensional outcome that cannot be inferred from limb size alone.

The present findings also highlight the clinical value of physical examination beyond objective staging and limb measurements. Shoulder ROM limitation was associated with higher pain intensity, worse QuickDASH scores, higher LYMQOL function, appearance, and symptom scores, and lower global quality-of-life scores. Unlike limb volume or circumference, shoulder mobility is directly related to upper-limb use during reaching, dressing, lifting, self-care, and other daily activities. Therefore, even when objective lymphedema severity is modest, shoulder ROM limitation may contribute substantially to functional disability and reduced quality of life.

This interpretation is consistent with previous studies showing that BCRL is associated not only with swelling but also with impaired shoulder mobility, upper-limb dysfunction, reduced strength, sensory–motor impairment, and poorer quality of life [30]. Similarly, Martins da Silva and Rezende reported that shoulder flexion, abduction, and external rotation limitations after breast cancer surgery were associated with poorer functional capacity and global quality of life, whereas the presence of lymphedema alone did not fully explain these outcomes [31]. Our cross-sectional design cannot establish whether shoulder ROM limitation is caused by lymphedema itself; it more plausibly reflects broader treatment-related shoulder morbidity, including surgical and postradiotherapy changes, that co-occurs with but cannot be distinguished from lymphedema-specific burden in this dataset, such as fibrosis or axillary cording, which were not directly measured.

The association between ROM limitation and pain in our study is also clinically relevant. Pain and restricted shoulder mobility may reinforce each other through reduced arm use, protective movement patterns, soft tissue tightness, axillary cording, treatment-related fibrosis, and fear of movement. Recent rehabilitation-focused evidence also supports the inclusion of pain, ROM, function, and quality-of-life outcomes alongside limb volume when evaluating nonpharmacological interventions for BCRL [32,33]. Pain was assessed as the general pain experienced in the lymphedematous limb over the preceding week; this measure does not distinguish the specific source of pain, such as neuropathy, postsurgical sensory changes, radiotherapy, rotator cuff pathology, or axillary cording. Our findings therefore reflect the overall pain burden perceived by patients in the affected limb. Accordingly, a comprehensive clinical evaluation for individuals with BCRL may benefit from considering pain, shoulder mobility, functional status, and patient-reported quality of life alongside edema reduction, rather than focusing on limb volume alone.

**Figure 3:**
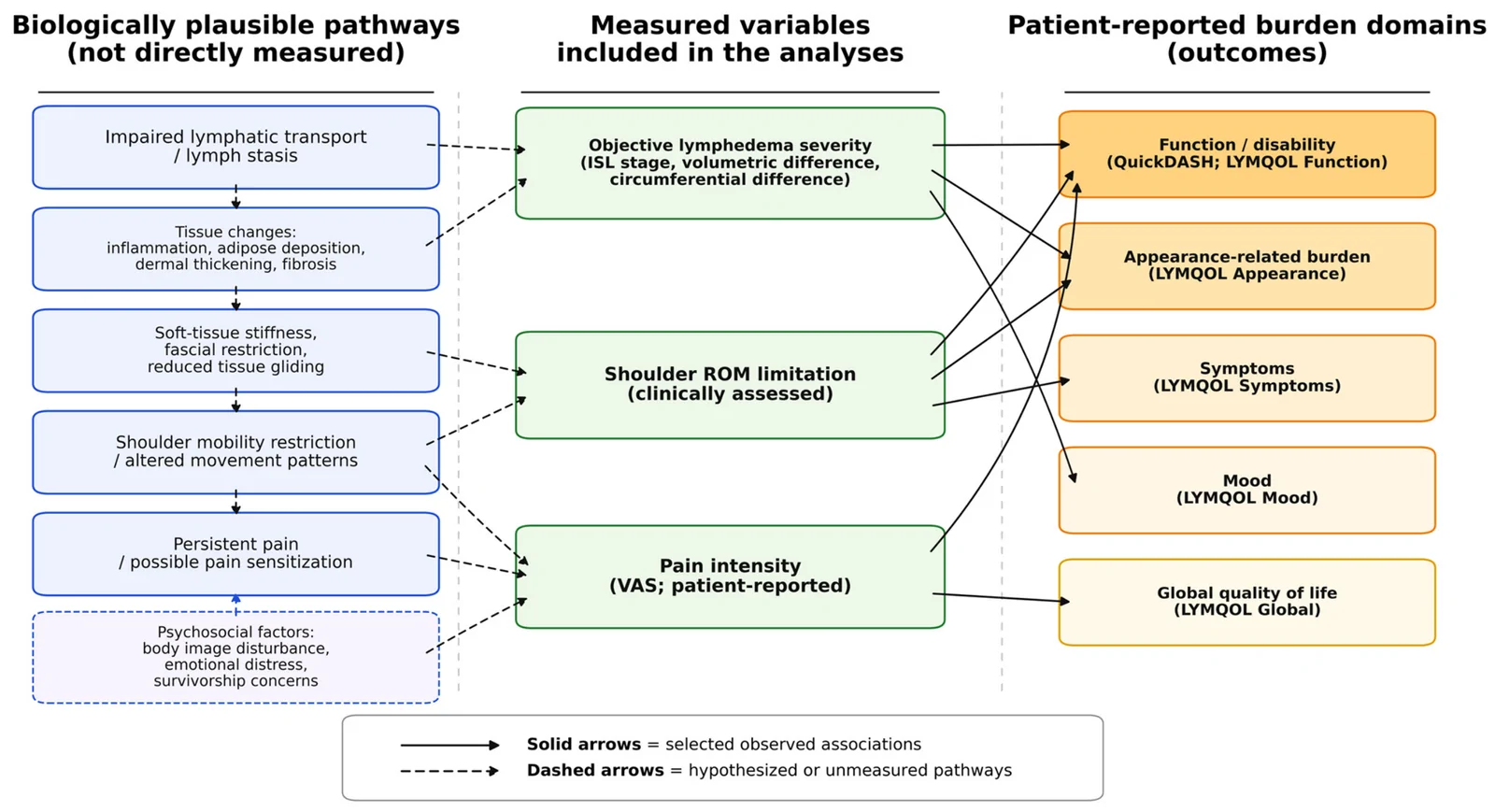
Proposed mechanistic framework linking objective lymphedema severity, shoulder ROM limitation, and pain intensity to patient-reported burden in BCRL. Solid arrows show selected observed associations in the present cross-sectional data, whereas dashed arrows show biologically plausible but unmeasured pathways. This framework does not imply causality. BCRL, breast cancer-related upper-limb lymphedema; ROM, range of motion; VAS, visual analog scale; LYMQOL, Quality of Life Measure for Limb Lymphedema; QoL, quality of life.

Beyond these clinical associations, the burden experienced by patients with BCRL likely reflects both tissue-level and psychosocial processes beyond limb enlargement. Impaired lymphatic transport may lead to lymph stasis, chronic inflammation, and progressive tissue fibrosis, which over time may increase soft-tissue stiffness and contribute to shoulder mobility restriction [9,14]. Pain may also arise from postsurgical soft-tissue changes and altered movement patterns. In parallel, persistent swelling, body image disturbance, and survivorship-related distress may amplify symptom perception, and central sensitization may contribute in some survivors with persistent pain [15,34]. However, the present study did not directly assess lymphatic flow, tissue fibrosis, fascial adhesion, central sensitization, or body image disturbance; these mechanisms should therefore be regarded as biologically plausible hypotheses rather than confirmed pathways (Figure 3).

The results concerning the Stemmer sign must be interpreted with caution. The Stemmer sign is primarily a clinical diagnostic finding that supports the presence of lymphedema and may reflect more established tissue changes rather than directly measuring patient-perceived burden [35]. In this study, participants with a positive Stemmer sign had worse QuickDASH, LYMQOL function, LYMQOL appearance, LYMQOL mood, and global quality-of-life scores in unadjusted comparisons, whereas pain intensity and LYMQOL symptom scores did not differ significantly according to Stemmer status. Because participants with a positive Stemmer sign also had substantially larger volumetric differences, these unadjusted associations are vulnerable to confounding by overall disease severity. After adjusting for volumetric difference, Stemmer sign remained associated with QuickDASH, LYMQOL function, LYMQOL mood, and global quality of life, whereas its associations with VAS, LYMQOL appearance, and LYMQOL symptom scores were attenuated to non-significance. This pattern suggests that Stemmer positivity may retain a distinct association with functional and psychosocial burden beyond overall severity, but it should not be interpreted as a direct marker of pain or symptom severity, and the unadjusted associations reported here should be regarded as crude estimates. The Stemmer sign is a useful clinical diagnostic sign for lymphedema, but narrative and clinical reviews have focused on its role in identifying lymphedema and supporting staging rather than measuring how much the patient feels the disease burden [35,36]. Likewise, observational data regarding lymphedema outcomes indicate that clinical signs and patient-reported outcomes yield complementary information and should be analyzed in conjunction [37]. Thus, in routine BCRL assessment, the Stemmer sign may help characterize the clinical phenotype of lymphedema, but it should be combined with objective limb measurements and PROMs to understand the full functional and quality-of-life impact.

The regression analyses further clarified the relative contribution of objective and clinically perceived factors to patient-reported outcomes. In the exploratory QuickDASH model, pain intensity, volumetric difference, and shoulder ROM limitation were statistically associated with upper-limb disability. This finding suggests that functional impairment in BCRL is shaped by both anatomical severity and clinically meaningful factors that directly affect arm use. Limb volume may reflect the physical burden of swelling, whereas pain and shoulder ROM limitation may more directly influence reaching, lifting, dressing, self-care, and other daily activities. This interpretation is consistent with previous studies reporting that upper-limb dysfunction after breast cancer treatment is closely related not only to lymphedema severity but also to shoulder mobility, pain, and broader treatment-related morbidity [12,30,31]. Because QuickDASH includes symptoms and functional difficulty, it conceptually overlaps with pain and shoulder ROM limitation. The findings from our multivariable model should therefore be interpreted as describing the statistical relationship among related dimensions of upper-limb dysfunction, rather than as evidence of distinct, independent causal contributions.

In contrast, the variables included in the model explained global quality of life only modestly. Pain intensity was the only variable statistically associated with global quality-of-life scores in this exploratory model, whereas volumetric difference was not significantly related to this outcome. This supports the view that global quality of life in individuals with BCRL is multidimensional and cannot be inferred from limb size alone. Previous studies and reviews have similarly emphasized that BCRL affects physical function, body image, psychological well-being, self-care, social participation, and long-term survivorship experiences [38,39,40,41]. Therefore, the modest explanatory value of the global quality-of-life model in the present study may reflect the influence of psychosocial and survivorship-related factors that were not fully captured in our dataset. Given the limited explanatory power of this exploratory model, these findings should not be interpreted as evidence that pain is the central determinant of global quality of life in this population.

Taken together, these findings indicate that objective staging and limb measurements are necessary for characterizing the anatomical severity of BCRL but are insufficient to capture the full patient-reported burden of the condition. Clinical examination findings, particularly shoulder ROM limitation and Stemmer sign, provide additional information on the functional, appearance-related, and psychosocial dimensions of BCRL. Recent evidence on nonpharmacological and physical therapy interventions also supports monitoring pain, ROM, function, and quality-of-life outcomes alongside limb volume and circumference [32,33]. Therefore, objective assessments should be complemented by PROMs and targeted clinical evaluation of pain, shoulder mobility, and relevant clinical signs when planning rehabilitation for individuals with BCRL.

These considerations have implications for rehabilitation, which is typically multimodal in BCRL, combining education, skin care, compression, exercise, and manual lymphatic drainage when indicated [42]. Progressive exercise programs are increasingly recognized as safe and beneficial for upper-limb function and quality of life [33]. However, our findings suggest that edema reduction alone is unlikely to address the full burden of BCRL; rehabilitation should also address pain, shoulder mobility, and lymphedema-specific quality of life alongside limb volume.

This study has several limitations. First, the cross-sectional design precludes causal interpretation of the observed associations, and the relationships among objective lymphedema severity, pain, shoulder ROM limitation, functional impairment, and quality of life should be interpreted as associations rather than determinants of outcome. In particular, we cannot determine whether increasing lymphedema severity leads to shoulder mobility restriction and pain, or whether postoperative shoulder morbidity contributes to both edema progression and patient-reported burden; longitudinal studies with repeated clinical and patient-reported assessments are needed to clarify this temporal relationship. Second, the dataset included detailed clinical and patient-reported variables; some factors that may influence patient-perceived burden, such as neuropathic pain features, psychological distress, body image, social support, self-care adherence, and detailed treatment-related sequelae, were not comprehensively assessed. Third, shoulder ROM limitation was analyzed as a dichotomous clinical variable rather than as direction-specific angular measurements. Therefore, the magnitude and pattern of ROM restriction could not be evaluated. Fourth, no a priori sample size calculation was performed for the present analysis, and the sample size was determined by the available consecutively enrolled cohort assessed during the study period. This limits the confirmatory strength of subgroup comparisons, correlation analyses, and multivariable models. In particular, the number of participants with Stage III lymphedema was small, which limits the interpretation of subgroup comparisons involving advanced-stage disease and means that findings involving advanced-stage lymphedema should be interpreted descriptively rather than generalized to the broader population with severe BCRL. Consistent with this, the sensitivity analysis combining Stage II and Stage III (Section 3.3) suggests that the absence of a significant difference between these two stages in the original three-group comparison more likely reflects limited statistical power than a true equivalence in disease burden. Fifth, objective assessment was based on clinical staging, circumferential measurements, and water displacement volumetry. Although bioimpedance spectroscopy, perometry, and imaging-based tissue assessment are valuable modalities for studies involving lymphedema patients, these methods were not available at our institution during the study period, and their retrospective addition was not possible given the cross-sectional design of the dataset. Future prospective studies incorporating these modalities would be a valuable direction for this field. Finally, the study included only women with breast cancer-related upper-limb lymphedema evaluated at a single center by the same physical medicine and rehabilitation specialist, which may limit the generalizability of the findings to other lymphedema populations, care settings, or examiners. Because clinical assessments were performed by a single examiner, intra-rater (test–retest) reliability was not formally assessed. In addition, radiotherapy and chemotherapy history were recorded only as present or absent; details such as radiation target volume and chemotherapy regimen, which may vary considerably between patients, were not captured and therefore could not be used for stratified analysis.

## 5. Conclusions

This study showed that objective severity indicators in BCRL were mainly associated with upper-limb functional impairment and appearance-related quality-of-life burden. However, pain, symptom burden, and global quality of life were not fully explained by lymphedema stage, limb volume, or mean circumferential difference. Pain intensity and shoulder ROM limitation appear to be clinically meaningful components of patient-reported burden.

These findings support a multidimensional clinical assessment approach in women with BCRL, combining objective severity measures with PROMs and targeted evaluation of pain and shoulder mobility. Such an approach may help capture aspects of patient-reported burden that limb measurements alone do not fully reflect.

## Figures and Tables

**Table 1:** Demographic and Clinical Characteristics of the Participants (*N* = 110).

Variable	Value
Age (years), mean ± SD	54.1 ± 10.0
Body mass index (kg/m^2^), mean ± SD	31.9 ± 5.8
Lymphedema duration (months), median (Q1–Q3)	9.0 (1.3–22.0)
Educational level, *n* (%)	
Literate	17 (15.4)
Primary school	53 (48.2)
Middle school	5 (4.5)
High school	26 (23.6)
University	7 (6.4)
Postgraduate	2 (1.8)
Employed, n (%)	10 (9.1)
Right dominant hand, n (%)	98 (89.1)
Current smoker, n (%)	11 (10.0)
Lymphedema side, n (%)	
Right	61 (55.5)
Left	48 (43.6)
Bilateral	1 (0.9)
Cancer stage, n (%)	
Stage I	5 (4.5)
Stage II	35 (31.8)
Stage III	33 (30.0)
Stage IV	11 (10.0)
Unknown	26 (23.6)
Lymphedema stage, n (%)	
Stage I	80 (72.7)
Stage II	25 (22.7)
Stage III	5 (4.5)
Lymph node dissection, n (%)	
Sentinel lymph node dissection	13 (11.8)
Axillary lymph node dissection	72 (65.5)
Unknown	25 (22.7)
Radiotherapy history, n (%)	93 (84.5)
Shoulder ROM limitation, n (%)	22 (20.0)
Positive Stemmer sign, n (%)	29 (26.4)
Pitting edema existence, n (%)	42 (38.2)
Axillary cording existence, n (%)	8 (7.3)
Motor deficit existence, n (%)	9 (8.2)

ROM; range of motion.

**Table 2:** Measurements and Patient-Reported Outcomes.

Variable	Median (Q1–Q3)
VAS	3.0 (0.0–4.0)
QuickDASH	22.7 (13.6–34.1)
LYMQOL Function	16.0 (14.0–20.8)
LYMQOL Appearance	8.0 (6.0–10.0)
LYMQOL Symptoms	11.0 (9.0–13.0)
LYMQOL Mood	9.0 (6.0–12.0)
Global Quality of Life	7.0 (6.0–9.0)
Volumetric Difference (mL)	337.5 (220.0–645.0)
Mean Circumferential Difference (cm)	1.4 (1.0–2.0)

VAS: Visual Analog Scale; QuickDASH; Quick Disabilities of the Arm, Shoulder and Hand questionnaire, LYMQOL; Lymphedema Quality of Life Tool.

**Table 3:** Comparison of Objective Measurements and Patient-Reported Outcomes According to Lymphedema Stage.

Variable	Stage I (n = 80)	Stage II (n = 25)	Stage III (n = 5)	*p* Value
VAS	3.0 (0.0–4.0)	3.0 (2.0–5.0)	3.0 (3.0–4.0)	0.737
QuickDASH	20.4 (13.0–31.8)	29.5 (18.2–40.9)	29.5 (22.7–47.7)	**0.020**
LYMQOL Function	15.0 (13.0–19.0)	20.0 (15.0–23.0)	22.0 (16.0–27.0)	**0.003**
LYMQOL Appearance	8.0 (6.0–9.0)	10.0 (10.0–12.0)	9.0 (9.0–13.0)	**<0.001**
LYMQOL Symptoms	11.0 (8.8–13.0)	11.0 (9.0–13.0)	12.0 (9.0–13.0)	0.530
LYMQOL Mood	8.0 (6.0–12.0)	12.0 (7.0–12.0)	11.0 (7.0–13.0)	0.268
Global Quality of Life	8.0 (7.0–9.0)	6.0 (6.0–8.0)	6.0 (5.0–8.0)	0.052
Mean Circumferential Difference (cm)	1.05 (0.90–1.50)	3.40 (2.10–4.40)	3.40 (2.80–5.80)	**<0.001**
Volumetric Difference (mL)	255 (210–354)	950 (720–1150)	1250 (1040–1250)	**<0.001**

Data are presented as median (Q1–Q3). *p* values were obtained using the Kruskal–Wallis test. Bold *p* values indicate statistical significance (*p* < 0.05). Stage III lymphedema included only five participants; therefore, findings for this subgroup should be interpreted with caution.

**Table 4:** Correlations Between Objective Lymphedema Severity Measures and Patient-Reported Outcomes.

Outcome	Lymphedema Stage ρ (*p*)	Volumetric Difference ρ (*p*)	Mean Circumferential Difference ρ (*p*)
VAS	0.074 (0.442)	−0.039 (0.687)	−0.038 (0.693)
QuickDASH	0.266 (0.005)	0.225 (0.018)	0.292 (0.002)
LYMQOL Function	0.322 (<0.001)	0.248 (0.009)	0.307 (0.001)
LYMQOL Appearance	0.416 (<0.001)	0.404 (<0.001)	0.456 (<0.001)
LYMQOL Symptoms	0.108 (0.262)	0.080 (0.408)	0.141 (0.142)
LYMQOL Mood	0.150 (0.117)	0.044 (0.648)	0.259 (0.006)
Global Quality of Life	−0.231 (0.015)	−0.093 (0.334)	−0.273 (0.004)

Data are presented as Spearman correlation coefficients (ρ) and *p* values.

**Table 5:** Associations of Clinical Examination Findings With Patient-Reported Outcomes.

Outcome	ROM Limitation Absent (n = 88)	ROM Limitation Present (n = 22)	*p* Value	Stemmer Negative (n = 81)	Stemmer Positive (n = 29)	*p* Value
VAS	3.0 (0.0–4.0)	4.0 (3.0–4.8)	0.005	3.0 (0.0–4.0)	3.0 (2.0–5.0)	0.155
QuickDASH	20.4 (9.1–31.8)	31.8 (24.3–50.0)	0.001	20.4 (9.1–27.3)	31.8 (25.0–47.7)	<0.001
LYMQOL Function	15.0 (13.0–18.3)	21.0 (19.3–23.8)	<0.001	15.0 (13.0–19.0)	21.0 (15.0–24.0)	<0.001
LYMQOL Appearance	8.0 (6.0–10.0)	10.0 (8.0–12.0)	0.009	8.0 (6.0–9.0)	10.0 (9.0–12.0)	<0.001
LYMQOL Symptoms	10.0 (9.0–12.0)	13.0 (11.0–13.8)	0.002	10.0 (9.0–13.0)	12.0 (9.0–13.0)	0.107
LYMQOL Mood	8.5 (6.0–12.0)	10.5 (7.0–13.0)	0.142	8.0 (6.0–12.0)	12.0 (9.0–13.0)	0.009
Global QoL	8.0 (6.0–9.0)	7.0 (5.0–7.8)	0.009	8.0 (7.0–9.0)	6.0 (5.0–7.0)	0.002

Data are presented as median (Q1–Q3). *p* values were obtained using the Mann–Whitney U test.

**Table 6:** Multivariable Linear Regression Models for Disability and Global Quality of Life.

Predictor	QuickDASH B	95% CI	Std. β	95% CI	*p*	VIF	Global QoL B	95% CI	Std. β	95% CI	*p*	VIF
VAS	3.026	1.811 to 4.241	0.404	0.242 to 0.567	**<0.001**	1.08	−0.186	−0.326 to −0.045	−0.249	−0.437 to −0.061	**0.010**	1.08
BMI	0.117	−0.379 to 0.613	0.041	−0.132 to 0.214	0.641	1.22	−0.006	−0.064 to 0.051	−0.022	−0.223 to 0.179	0.829	1.22
Age	−0.166	−0.450 to 0.117	−0.101	−0.272 to 0.071	0.247	1.19	−0.005	−0.037 to 0.028	−0.028	−0.227 to 0.170	0.777	1.19
Volumetric difference	0.012	0.005 to 0.019	0.265	0.104 to 0.426	**0.001**	1.06	−0.0004	−0.001 to 0.0004	−0.089	−0.276 to 0.098	0.345	1.06
ROM limitation	8.666	1.513 to 15.818	0.210	0.037 to 0.383	**0.018**	1.23	−0.744	−1.572 to 0.083	−0.181	−0.382 to 0.020	0.077	1.23

B: unstandardized regression coefficient; CI: confidence interval; β: standardized regression coefficient; VIF: variance inflation factor; ROM: range of motion; QoL: quality of life. The QuickDASH model explained 35.2% of the variance in disability scores (adjusted R^2^ = 0.321), whereas the global quality-of-life model explained 12.7% of the variance in global quality-of-life scores (adjusted R^2^ = 0.085). Bold p values indicate statistical significance (*p* < 0.05).

## Data Availability

The raw data supporting the conclusions of this article will be made available by the authors on request.

## Supplementary Materials

The following supporting information can be downloaded at: https://www.mdpi.com/article/10.3390/jcm15166345/s1, Table S1: Raw and Benjamini–Hochberg false discovery rate-adjusted *p*-values for the main bivariate analyses; Table S2: Sensitivity analyses comparing ISL Stage I with combined ISL Stages II–III; Table S3: Subgroup Comparison by Lymph Node Dissection Type; Table S4: Comparison by Radiotherapy History.

## Abbreviations

The following abbreviations are used in this manuscript:

ISL	International Society of Lymphology
LYMQOL	Quality of life measure for limb lymphoedema
BCRL	Breast cancer-related upper-limb lymphedema
ROM	Range-of-motion
QuickDASH	Quick Disabilities of the Arm, Shoulder and Hand questionnaire
