# Supplementary material for: Objective Severity, Pain and Range-of-Motion Limitation in Breast Cancer-Related Upper-Limb Lymphedema: Associations with Patient-Reported Burden
Source: J Clin Med. 2026 Aug 17;15(16):6345. doi: 10.3390/jcm15166345 (PMC13513956; doi:10.3390/jcm15166345)
Supplement: Supplementary file 1 [file jcm-15-06345-s001.zip › jcm-4470811-supplementary.pdf]

Supplementary Materials *Manuscript ID: jcm-4470811*

Table S1. Raw and FDR-Adjusted p-Values for Primary Analyses (Tables 3–5)

Method: Benjamini-Hochberg false discovery rate (FDR) correction applied across the 44 statistical tests comprising the primary analyses reported in Tables 3, 4, and 5 of the main manuscript. Rows shaded green indicate comparisons that remained statistically significant (adjusted p<0.05) after correction.

Source Table	Comparison	Spearman ρ	Raw p	FDR-Adjusted p
Table 3	VAS	—	0.737	0.737
Table 3	QuickDASH	—	0.020	0.031
Table 3	LYMQOL Function	—	0.003	0.009
Table 3	LYMQOL Appearance	—	<0.001	<0.001
Table 3	LYMQOL Symptoms	—	0.530	0.583
Table 3	LYMQOL Mood	—	0.268	0.327
Table 3	Global QoL	—	0.052	0.079
Table 3	Mean Circumferential Difference	—	<0.001	<0.001
Table 3	Volumetric Difference	—	<0.001	<0.001
Table 4	Lymphedema Stage x VAS	0.074	0.443	0.500
Table 4	Lymphedema Stage x QuickDASH	0.266	0.005	0.011
Table 4	Lymphedema Stage x LYMQOL Function	0.322	<0.001	0.002
Table 4	Lymphedema Stage x LYMQOL Appearance	0.416	<0.001	<0.001
Table 4	Lymphedema Stage x LYMQOL Symptoms	0.108	0.263	0.327
Table 4	Lymphedema Stage x LYMQOL Mood	0.15	0.118	0.167
Table 4	Lymphedema Stage x Global QoL	-0.231	0.015	0.026
Table 4	Volumetric Difference x VAS	-0.039	0.685	0.712
Table 4	Volumetric Difference x QuickDASH	0.225	0.018	0.029
Table 4	Volumetric Difference x LYMQOL Function	0.248	0.009	0.016
Table 4	Volumetric Difference x LYMQOL Appearance	0.404	<0.001	<0.001
Table 4	Volumetric Difference x LYMQOL Symptoms	0.08	0.404	0.468
Table 4	Volumetric Difference x LYMQOL Mood	0.044	0.650	0.697
Table 4	Volumetric Difference x Global QoL	-0.093	0.336	0.400
Table 4	Mean Circumferential Difference x VAS	-0.038	0.696	0.712
Table 4	Mean Circumferential Difference x QuickDASH	0.292	0.002	0.006
Table 4	Mean Circumferential Difference x LYMQOL Function	0.307	0.001	0.004
Table 4	Mean Circumferential Difference x LYMQOL Appearance	0.456	<0.001	<0.001
Table 4	Mean Circumferential Difference x LYMQOL Symptoms	0.141	0.141	0.190

Source Table	Comparison	Spearman ρ	Raw p	FDR-Adjusted p
Table 4	Mean Circumferential Difference x LYMQOL Mood	0.259	0.006	0.013
Table 4	Mean Circumferential Difference x Global QoL	-0.273	0.004	0.010
Table 5	ROM Limitation - VAS	—	0.005	0.011
Table 5	ROM Limitation - QuickDASH	—	0.001	0.005
Table 5	ROM Limitation - LYMQOL Function	—	<0.001	<0.001
Table 5	ROM Limitation - LYMQOL Appearance	—	0.009	0.016
Table 5	ROM Limitation - LYMQOL Symptoms	—	0.002	0.006
Table 5	ROM Limitation - LYMQOL Mood	—	0.142	0.190
Table 5	ROM Limitation - Global QoL	—	0.009	0.016
Table 5	Stemmer Sign - VAS	—	0.155	0.200
Table 5	Stemmer Sign - QuickDASH	—	<0.001	<0.001
Table 5	Stemmer Sign - LYMQOL Function	—	<0.001	0.002
Table 5	Stemmer Sign - LYMQOL Appearance	—	<0.001	<0.001
Table 5	Stemmer Sign - LYMQOL Symptoms	—	0.107	0.157
Table 5	Stemmer Sign - LYMQOL Mood	—	0.009	0.016
Table 5	Stemmer Sign - Global QoL	—	0.002	0.006

All comparisons significant at the raw p<0.05 threshold remained significant after FDR correction; no findings were lost.

Table S2. Sensitivity Analysis: Stage I versus Combined Stage II+III

Method: Mann-Whitney U test. Stage II (n=25) and Stage III (n=5) were combined into a single group (n=30) and compared with Stage I (n=80), to evaluate whether the small Stage III subgroup limited statistical power in the original three-group comparison.

Variable	Stage I Median (Q1–Q3)	Stage II+III Median (Q1–Q3)	p (2-group)	p (original 3-group)
VAS	3.0 (0.0-4.0)	3.0 (2.0-4.8)	0.455	0.737
QuickDASH	20.4 (9.1-31.8)	29.5 (19.3-46.0)	0.005	0.020
LYMQOL Function	15.0 (13.0-19.0)	20.5 (15.0-23.8)	<0.001	0.003
LYMQOL Appearance	8.0 (6.0-9.0)	10.0 (9.0-12.8)	<0.001	<0.001
LYMQOL Symptoms	11.0 (8.8-13.0)	11.5 (9.0-13.0)	0.262	0.530
LYMQOL Mood	8.0 (6.0-12.0)	11.5 (7.0-12.8)	0.109	0.268
Global QoL	8.0 (7.0-9.0)	6.0 (5.2-8.0)	0.015	0.052
Mean Circumferential Difference	1.1 (0.9-1.5)	3.4 (2.1-4.6)	<0.001	<0.001
Volumetric Difference	255.0 (210.0-353.8)	975.0 (752.8-1172.5)	<0.001	<0.001

Global quality of life, which was not significant in the original three-group comparison (p=0.052), became statistically significant in the two-group sensitivity analysis (p=0.015).

Table S3. Subgroup Comparison by Lymph Node Dissection Type

Method: Mann-Whitney U test. Sentinel lymph node dissection (SLND, n=13) versus axillary lymph node dissection (ALND, n=72); participants with unknown dissection type (n=25) were excluded from this comparison (n=85 analyzed).

Variable	SLND Median (Q1–Q3)	ALND Median (Q1–Q3)	p
VAS	3.0 (0.0-4.0)	3.0 (0.0-4.0)	0.925
QuickDASH	24.0 (6.8-29.5)	21.6 (9.1-34.3)	0.479
LYMQOL Function	16.0 (14.0-21.0)	15.0 (13.0-20.2)	0.611
LYMQOL Appearance	7.0 (5.0-10.0)	8.0 (6.8-10.0)	0.293
LYMQOL Symptoms	10.0 (9.0-12.0)	11.0 (9.0-13.0)	0.376
LYMQOL Mood	7.0 (6.0-10.0)	9.0 (6.0-13.2)	0.230
Global QoL	8.0 (6.0-9.0)	7.0 (6.0-9.0)	0.341
Mean Circumferential Difference	1.2 (0.9-1.7)	1.4 (0.9-1.7)	1.000
Volumetric Difference	330.0 (235.0-450.0)	305.0 (215.0-530.0)	0.760

No statistically significant differences were observed between SLND and ALND for any variable (all p>0.29).

Table S4. Comparison by Radiotherapy History

Method: Mann-Whitney U test. Participants with (n=93) versus without (n=17) a history of radiotherapy.

Variable	No Radiotherapy Median (Q1–Q3)	Radiotherapy Median (Q1–Q3)	p
VAS	3.0 (0.0-4.0)	3.0 (0.0-4.0)	0.670
QuickDASH	27.2 (20.4-36.4)	22.7 (11.4-34.1)	0.181
LYMQOL Function	18.0 (14.0-22.0)	16.0 (13.0-20.0)	0.311
LYMQOL Appearance	8.0 (7.0-9.0)	8.0 (6.0-10.0)	0.573
LYMQOL Symptoms	10.0 (8.0-13.0)	11.0 (9.0-13.0)	0.980
LYMQOL Mood	12.0 (9.0-15.0)	8.0 (6.0-12.0)	0.021
Global QoL	7.0 (5.0-7.0)	8.0 (6.0-9.0)	0.006
Mean Circumferential Difference	1.2 (0.8-1.6)	1.4 (1.0-2.1)	0.164
Volumetric Difference	235.0 (210.0-340.0)	350.0 (225.0-650.0)	0.068

No significant differences were found in lymphedema stage, QuickDASH, volumetric difference, or mean circumferential difference (all p>0.06). LYMQOL Mood and global quality-of-life scores differed significantly according to radiotherapy history.
